# Thermotropic phase behavior and headgroup interactions of the nonbilayer lipids phosphatidylethanolamine and monogalactosyldiacylglycerol in the dry state

**DOI:** 10.1186/2046-1682-4-11

**Published:** 2011-05-10

**Authors:** Antoaneta V Popova, Dirk K Hincha

**Affiliations:** 1 Max-Planck-Institut für Molekulare Pflanzenphysiologie, Am Mühlenberg 1, D-14476 Potsdam, Germany; 2 Institute of Biophysics, Bulgarian Academy of Sciences, 1113 Sofia, Bulgaria

## Abstract

**Background:**

Although biological membranes are organized as lipid bilayers, they contain a substantial fraction of lipids that have a strong tendency to adopt a nonlamellar, most often inverted hexagonal (H_II_) phase. The polymorphic phase behavior of such nonbilayer lipids has been studied previously with a variety of methods in the fully hydrated state or at different degrees of dehydration. Here, we present a study of the thermotropic phase behavior of the nonbilayer lipids egg phosphatidylethanolamine (EPE) and monogalactosyldiacylglycerol (MGDG) with a focus on interactions between the lipid molecules in the interfacial and headgroup regions.

**Results:**

Liposomes were investigated in the dry state by Fourier-transform Infrared (FTIR) spectroscopy and Differential Scanning Calorimetry (DSC). Dry EPE showed a gel to liquid-crystalline phase transition below 0°C and a liquid-crystalline to H_II _transition at 100°C. MGDG, on the other hand, was in the liquid-crystalline phase down to -30°C and showed a nonbilayer transition at about 85°C. Mixtures (1:1 by mass) with two different phosphatidylcholines (PC) formed bilayers with no evidence for nonbilayer transitions up to 120°C. FTIR spectroscopy revealed complex interactions between the nonbilayer lipids and PC. Strong H-bonding interactions occurred between the sugar headgroup of MGDG and the phosphate, carbonyl and choline groups of PC. Similarly, the ethanolamine moiety of EPE was H-bonded to the carbonyl and choline groups of PC and probably interacted through charge pairing with the phosphate group.

**Conclusions:**

This study provides a comprehensive characterization of dry membranes containing the two most important nonbilayer lipids (PE and MGDG) in living cells. These data will be of particular relevance for the analysis of interactions between membranes and low molecular weight solutes or soluble proteins that are presumably involved in cellular protection during anhydrobiosis.

## Background

Biological membranes are composed of a wide range of lipids with different physicochemical properties. While the major components are usually bilayer forming lipids, most cellular membranes also contain a significant complement of lipids that can adopt nonbilayer structures such as an inverted hexagonal phase (H_II_) [1,2]. Some biologically important functions such as membrane fusion [3,4] are related to formation of H_II _structures. Therefore, the formation of H_II _has been the subject of considerable interest and different molecular models have been developed that reflect the physical and chemical properties governing the transition from lamellar to H_II _phase [5-7]. The transition dependends on different internal (molecular shape, degree of unsaturation and length of the fatty acyl chains, charge and hydration of the headgroup) and external factors (water content, temperature, pH, ionic strength, solutes) [6-10]. In addition, H-bonding between lipid molecules also contributes to their phase behavior [11].

Cellular membranes are primary sites of damage during environmental stresses such as freezing or drying. In addition, membrane stability can be compromised during technical biopreservation procedures such as cell or organ cryopreservation, or the preparation of dry biomaterials for long-term storage. Liposomes have been used for many years as convenient model systems to study the effects of membrane lipid composition and soluble protectants such as sugars on membrane stability during drying in the context of anhydrobiosis in microbes, animals and plants, and the development of improved biopreservation protocols (see [12-14] for reviews).

Lipid composition, especially the presence of the nonbilayer lipids phosphatidylethanolamine (PE) and monogalactosyldiacylglycerol (MGDG), can strongly influence membrane stability during dehydration and the efficiency of amphiphilic small molecules [15-17] and soluble plant stress proteins [18,19] to stabilize membranes. It is therefore important to investigate the phase behavior and interactions between lipids in dry membranes containing either only nonbilayer lipids or mixtures of nonbilayer and bilayer lipids to better understand the effects of such protectants on membrane structure and their interactions with the membrane surface. Anhydrous systems are of particular utility for such studies, as they allow the unambiguous identification of molecular interactions without the interfering effects of water. Obviously, once the anhydrous system has been thoroughly characterized, it will be possible to systematically investigate the contributions of small amounts of water in partitially dehydrated samples, as they will often occur under natural conditions.

PE is one of the most abundant lipids in cellular membranes and can form either lamellar or nonlamellar phases. Increasing temperatures lead to a transition from the gel to the liquid-crystalline phase, followed by a transition to H_II _in the fully hydrated state. However, also direct gel to H_II _transitions without an intermediate liquid-crystalline phase have been reported [2,7]. Differential Scanning Calorimetry (DSC) experiments have shown that the transition from gel to liquid-crystalline state is highly cooperative and strongly endothermic, while the transition from liquid-crystalline to H_II _has a low enthalpy [7] indicating that the acyl chains of the lipids in nonbilayer conformation are not markedly more disordered than in the liquid-crystalline state of the bilayer [20].

Mixing PE with bilayer lipids such as phosphatidylcholine (PC) favours the formation of bilayers [1]. The stability of these mixed bilayers depends on the ratio between bilayer and nonbilayer lipids [20-22]. Generally, it is assumed that the ability of PC to stabilize bilayers containing H_II _lipids is a consequence of the phase preference of PC rather than the result of specific interactions between PC and PE [2].

The most abundant polar lipid on Earth is MGDG. It comprises about 50% of the total lipid content of photosynthetic membranes in higher plants and algae and is, like PE, a nonbilayer lipid [23-25]. Hydrated MGDG from the leaves of higher plants forms nonbilayer phases over a wide temperature range of approximately -15°C to 80°C [26-28]. Similar to PE, MGDG can be stabilized in lamellar structures when mixed with PC [29,30].

H_II _formation in membranes can be induced by dehydration, either through the removal of water or the additon of osmolytes [22,31-36]. These reports concern mainly the phase behavior of lipids at different degrees of hydration. However, no studies have specifically focused on mixed membranes composed of bilayer and nonbilayer lipids in the dry state and in particular on the interactions between the lipids. We therefore investigated the thermotropic phase behavior of dry membranes containing egg PE (EPE) or MGDG by FTIR spectroscopy and DSC. The focus of our work was on the FTIR studies that allowed us to investigate the interactions between the different lipids to evaluate the contributions of headgroup interactions to the phase behavior of dry membranes.

## Methods

### Liposome preparation

Egg PC (EPC, fatty acid composition: 32.7% 16:0, 12.3% 18:0, 32.0% 18:1, 17.1% 18:2) and 1,2 dimyristoyl-sn-glycero-3-phosphocholine (DMPC, 100% 14:0) were obtained from Avanti Polar Lipids (Alabaster, AL, http://www.avantilipids.com). The chloroplast galactolipid MGDG (fatty acid composition: 21.5% 16:0, 5.7% 18:0, 6.0% 18:1, 64.5% 18:3) from spinach leaves and EPE (fatty acid composition: 22.0% 16:0, 37.4% 18:0, 29.4% 18:1, 11.2% 18:2) were purchased from Lipid Products (Redhill, Surrey, UK, http://lipidproducts.virtualave.net).

To make the results from the present study comparable to studies on the effects of various solutes and proteins on membranes in the dry state, we used extruded liposomes wherever possible. Lipids (10 mg of pure lipids or binary mixtures at a 1:1 mass ratio) were dried from chloroform under a stream of N_2 _and subsequently under vacuum over night. The resulting lipid film was hydrated in 200 μl of distilled water and liposomes were formed using a hand-held extruder (Avestin, Ottawa, Canada) with two layers of 100 nm pore filters [37].

### Fourier-transform infrared (FTIR) spectroscopy

Liposomes (50 μl) were spread on CaF_2 _windows and dried at 0% relative humidity in desiccators at 28°C for 6 to 8 hours in the dark and then kept over night under vacuum. Since pure EPE or MGDG do not form bilayers, 2.5 mg lipids in chloroform were spread directly on CaF_2 _windows, the solvent was evaporated under a stream of N_2 _and then under vacuum over night. FTIR measurements were performed with a Perkin-Elmer GX 2000 spectrometer as described in detail previously [38,39]. A window with the dry sample was placed in a cuvette holder, fixed in a vacuum chamber connected to a temperature control unit (Specac Eurotherm, Worthington, UK) and placed in the infrared beam. Temperature was monitored by a fine thermocouple fixed on the window next to the sample. Samples were kept in the sample holder under vacuum at 30°C for 30 min to remove residual moisture absorbed during handling. This was verified by the absence of a water band in the FTIR spectra at 1650 cm^-1^. In addition, the position of the νP=Oas band of PC above 1260 cm^-1 ^indicated that the samples were essentially anhydrous [40]. Temperature was then decreased to -30°C and after 10 min equilibration, the temperature was increased at a rate of 1°C min^-1^. Two spectra were recorded and coadded every minute. Spectra were analyzed using Spectrum 5.0.1 software. After normalization of absorbance and baseline correction of the spectra by the interactive abex and flat routines, the wavenumbers of the symmetric CH_2 _stretching vibration (νCH_2_s), C=O stretching vibration (νC=O), asymmetric P=O stretching vibration (νP=Oas), sugar OH stretching vibration (νOH) and asymmetric choline stretching vibration (νN^+^(CH_3_)_3_as) bands were determined by the peak identification routine. These peaks cover all relevant functional groups in the investigated lipids. The gel to liquid-crystalline phase transition temperature (T_m_) was determined as the midpoint of the shift in νCH_2_s with temperature [41]. The carbonyl stretching peak (νC=O) between 1770 cm^-1 ^and 1700 cm^-1 ^was analyzed by peak deconvolution and curve fitting using the peak-fitting module of OriginPro 7.0 as described in detail previously [40,42]. Correlation coefficients for all fitted curves were higher than 0.999.

### Differential scanning calorimetry (DSC)

Liposomes (50 μl) or 2.5 mg of EPE or MGDG in chloroform were applied to aluminium pans and dried as described for the FTIR samples. Earlier measurements had shown that such samples contained less than 0.02 g H_2_O/g lipid [40]. Pans were sealed in a glove bag under N_2 _atmosphere and transferred directly into a Netzsch (Selb, Germany) DSC 204. Samples were cooled to -70°C and then heated to 120°C with 5 min of equilibration at the lowest and highest temperature. Cooling and heating scans were performed three times at a rate of 20°C min^-1 ^with each sample and at least three samples were measured. Analysis of the thermograms was performed using the Netzsch software package. The phase transition temperatures (gel to liquid-crystalline (T_m_) or bilayer to nonbilayer (T_hex_)) were determined as the temperature at the peak maximum of the second heating scan. The enthalpy change of the phase transitions (ΔH) was obtained from the areas under the peaks of the transitions.

## Results

### Phase behavior of the dry lipids

**Figure 1:**
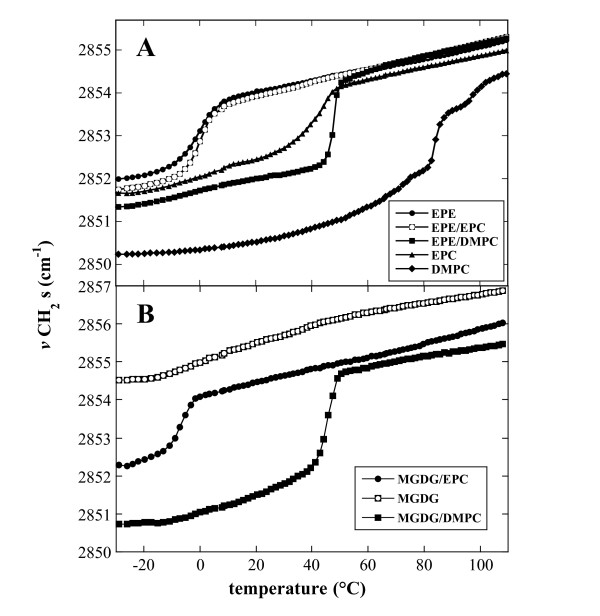
**Lipid melting curves determined as the temperature dependent increase in the position of the CH_2 _symmetric stretching vibration band (νCH_2_s) of the fatty acyl chains in FTIR spectra**. Samples contained either the pure lipids or binary lipid mixtures at a 1:1 mass ratio as indicated in the panels. Phase transition temperatures (T_m_) were determined as the midpoints of the melting curves and are shown in Table 1.

Figure 1 shows the melting curves of dry EPE and MGDG and of the respective 1:1 mixtures of the two nonbilayer lipids with either EPC or DMPC, as determined by FTIR spectroscopy. For comparison the melting curves of dry liposomes containing pure EPC or DMPC are also included in Figure 1A. The temperature induced increase in νCH_2_s indicates the increase in conformational disorder of the hydrocarbon chains associated with chain melting [43].

As expected for a fully saturated lipid, dry DMPC liposomes showed the highest T_m _(84°C) of all investigated samples. The T_m _of dry EPC, which mainly contains monounsaturated POPC, was more than 40°C lower, in agreement with our earlier measurements [39]. The T_m _of dry EPE was again almost 40°C lower than that of EPC. Interestingly, while the T_m _of a 1:1 mixture of EPE and DMPC was at the mid-point between the T_m _values of the pure lipids, a mixture of EPE and EPC showed the same melting behavior as pure EPE (Figure 1A). The νCH_2_s of all lipid samples except for DMPC were very similar, while DMPC showed consistently lower values, especially in the gel state, indicating tighter chain packing of saturated fatty acids.

νCH_2_s of MGDG was above 2854 cm^-1 ^at all temperatures with a gradual increase during heating, indicating a high degree of disorder typical for both the liquid-crystalline and nonbilayer phases (Figure 1B). Since these phases are characterized by a high degree of disorder in the fatty acid chains, they can not be distinguished by FTIR [44]. Binary mixtures of MGDG with EPC or DMPC showed well defined phase transitions, presumably from gel to liquid-crystalline state. In the gel phase νCH_2_s of MGDG/EPC was higher than for MGDG/DMPC liposomes in accordance with the differences in unsaturation.

For all melting curves in Figure 1, except for MGDG, the values of νCH_2_s at low temperatures are indicative of lipids in the gel phase. At high temperatures νCH_2_s-values could either indicate lipids in the liquid-crystalline or in a nonlamellar phase. In addition, liquid-crystalline to nonlamellar phase transitions may have occurred that were not detectable by FTIR.

**Figure 2:**
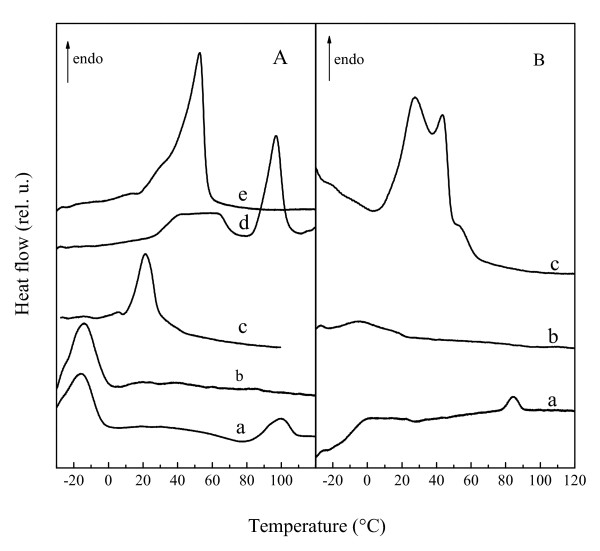
**DSC heating thermograms of dry samples**. Panel (A) shows data for pure EPE (a) and for dry liposomes containing 50% EPE/50% EPC (b), pure EPC (c), pure DMPC (d) and 50% EPE/50% DMPC (e). Panel (B) shows data for dry pure MGDG (a) and for dry liposomes containing 50% MGDG/50% EPC (b) and 50% MGDG/50% DMPC (c). Thermograms are from the second heating scan from -30°C to 120°C. Phase transition temperatures (T_m _and T_hex_) and transition enthalpies (ΔH) are shown in Table 1.

DSC measurements were performed to obtain a more complete picture of the phase behavior of the different lipids in the dry state (Figure 2). Fully hydrated PE and MGDG tend to arrange in nonlamellar structures such as cubic or H_II _phase, depending on temperature and fatty acid composition [9,26,45-48]. While in the fully hydrated state transitions often proceed from liquid-crystalline through a cubic intermediate to the H_II _phase, reduced water content favours a direct transition from the lamellar to the H_II _phase [49].

In the dry state EPE showed two phase transitions, the first at -15°C corresponding to the chain melting transition from gel to liquid-crystalline, followed by a low enthalpy transition at 100°C, attributed to a bilayer to H_II _transition (Figure 2A). For dry MGDG only one transition with low enthalpy at 85°C was observed (Figure 2B). These two thermograms indicate that EPE proceeded from gel through liquid-crystalline to H_II _phase during heating, while dry MGDG was only in either the liquid-crystalline or nonbilayer phase in the investigated temperature range. In all other samples, only transitions from gel to liquid-crystalline phase were observed. The transition temperatures and associated enthalpies are shown in Table 1 in comparison to the corresponding T_m _values determined by FTIR. The transition temperatures determined by DSC were lower than those determined by FTIR. We attribute this to residual water the samples may have absorbed during handling. Such water was removed in the FTIR experiments because the samples were kept under vacuum before and during the measurements. These differences, however, were consistent between different lipid samples and do not invalidate our conclusions regarding the lipid phase behavior.

**Table 1:** Effects of composition on the lipid melting behavior.

Sample composition	T_m _(°C) (FTIR)	T_m _(°C) (DSC)	T_hex _(°C) (DSC)	ΔH T_m_ (kJ/mol)	ΔH T_hex_ (kJ/mol)
100% EPE	-3.4	-14.9	100.4	10.30	3.79

50% EPE/50% EPC	0.0	-14.0		16.67	

50% EPE/50% DMPC	47.5	53.0		32.03	

100% EPC	39.6	22.0		27.20	

100% DMPC	84.3	97.6		23.20	

100% MGDG			84.7		0.96

50% MGDG/50% EPC	-9.0				

50% MGDG/50% DMPC	44.5	27.6/43.6		36.70	

Values for phase transition temperatures (T_m_) were either determined by FTIR (Fig. 1) or DSC (Fig. 2). Bilayer to hexagonal phase transition temperatures (T_hex_) and transition enthalpies (ΔH) were determined by DSC.

### H-bonding interactions of the carbonyl ester groups

The carbonyl ester (C=O) groups of diacylglycerolipids reside in the interfacial region of the lipid bilayer and are potential participants in H-bonding interactions with water or with other functional groups of the same or different lipid molecules. The C=O groups give rise to a major peak in the infrared spectra of lipids at around 1738 cm^-1 ^that is sensitive to the polarity of and interactions with the local environment. The C=O peak can be resolved into two underlying peaks. The higher wavenumber component band at 1742 cm^-1 ^arises from free (i.e. not H-bonded) C=O groups, while the downfield component band at 1726 cm^-1 ^originates from C=O groups involved in H-bonding [50-52]. The analysis of C=O peaks was performed either at 90°C (all lipid compositions except pure DMPC) or at 100°C (pure DMPC) as at these temperatures the lipids were in a stable liquid-crystalline state.

**Figure 3:**
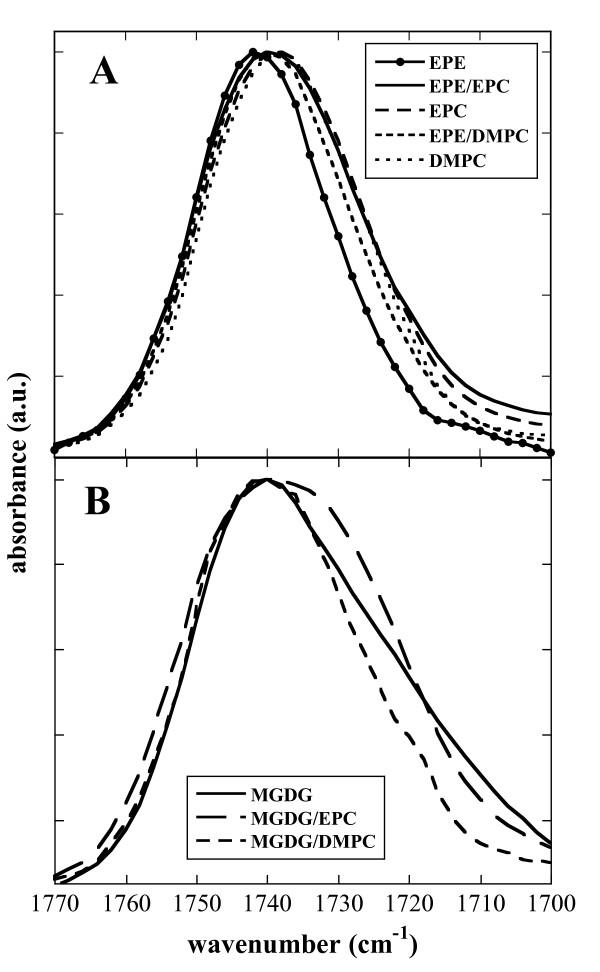
**Infrared spectra in the carbonyl stretching region**. Spectra were taken from dry EPE and from dry liposomes containing pure EPC, pure DMPC, 50% EPE/50% EPC and 50% EPE/50% DMPC (A) and from dry MGDG and from dry liposomes containing pure EPC, pure DMPC, 50% MGDG/50% EPC and 50% MGDG/50% DMPC (B). Spectra were measured at 90°C, except for DMPC at 100°C.

Figure 3 shows the C=O contours of all dry samples that were also analyzed for their melting behavior (Figures 1 and 2). The C=O contour of pure EPE is centered at 1740 cm^-1^, while all other peaks are located several wavenumbers lower. The peak measured with pure MGDG is broadened on the downfield side and those from liposomes containing MGDG/EPC or MGDG/DMPC show indications of component peaks attributable to free and H-bonded C=O (Figure 3B).

**Figure 4:**
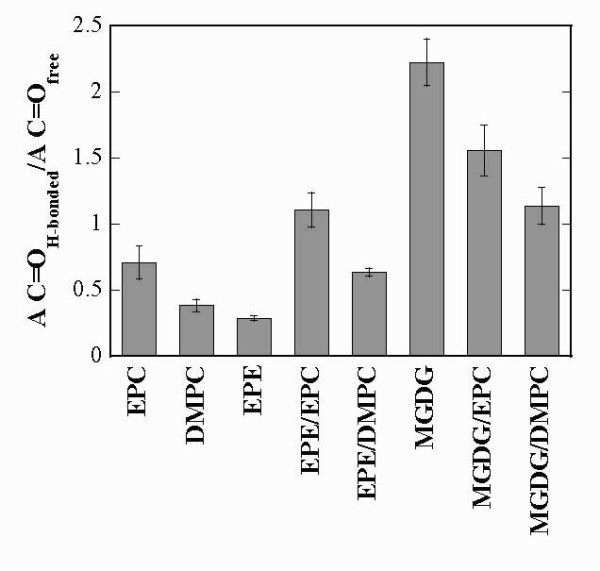
**Ratio between the fitted peak areas of the two component bands of the carbonyl stretching peaks shown in Fig. 3**. The ratio A C=O_H-bonded_/A C=O_free_, indicating the relative amount of H-bonding to the lipid C=O groups, is shown for the pure lipids and all binary mixtures. The values represent the means ± SE from at least 3 different samples.

For a quantitative analysis of the interactions of the C=O groups, the peak was deconvoluted into its two component bands centered at 1742 cm^-1 ^and 1728 cm^-1^. The relative area of the band components is proportional to the number of free and H-bonded C=O groups [40,42,53]. The ratio of the two component bands changes with changes in lipid composition. Since in our FTIR experiments the lipids are essentially anhydrous, the H-bonding observed in all samples must derive almost exclusively from interactions between different lipid molecules. For dry EPC liposomes the ratio A C=O_H-bonded_/A C=O_free _was 0.7 (Figure 4), indicating that the population of free C=O groups was higher than that of H-bonded C=O groups. We have presented evidence previously [40] that the most likely H-bonding interaction partners for C=O groups in dry EPC membranes are the choline groups of neighbouring lipid molecules. In dry DMPC H-bonding to C=O groups was less pronounced than in EPC due to the tighter packing of lipids containing only saturated acyl chains.

The ethanolamine headgroup of EPE is strongly involved in H-bonding with P=O groups ([9,54] and our results below) and is therefore shielded from the C=O groups that are situated deeper in the bilayer. Consequently, the ratio A C=O_H-bonded_/A C=O_free _for EPE was even lower than that for DMPC. In EPE/EPC liposomes the ratio of H-bonded to free C=O groups was increased compared to both pure lipids, indicating a shift of the pattern of H-bonding of ethanolamine groups away from the P=O and towards the C=O groups (compare also the analysis of the P=O vibrations below). The same was true for EPE/DMPC, but here the ratio was lower than for EPE/EPC (0.5 compared to 1.1), again indicating reduced interactions due to packing constraints.

The OH groups of the galactose residues in MGDG interacted with the C=O groups, resulting in a high ratio of H-bonded to free C=O groups for MGDG (Figure 4). In MGDG/EPC and MGDG/DMPC bilayers this ratio was decreased, due to the lower amount of galactose headgroups. In addition, the lower ratio in MGDG/DMPC compared to MGDG/EPC again indicated a reduction in H-bonding to C=O groups due to tighter packing of the lipids in membranes containing a fully saturated lipid.

### The headgroup region of the phospholipids and of MGDG

The hydrophilic part of the phospholipids used in this study contains the phosphate (P=O) and choline (N^+^(CH_3_)_3_) or ethanolamine (N^+^CH_3_) groups. The P=O group is situated above the C=O and gives rise to an asymmetric vibration in the 1300-1200 cm^-1 ^region of the infrared spectrum. While this frequency is located at 1262 cm^-1 ^in anhydrous PC, it can be shifted to around 1220 cm^-1 ^upon addition of water or sugars [17,40,52,55]. The extent of the shift is an indicator for the number and strength of the H-bonds.

**Figure 5:**
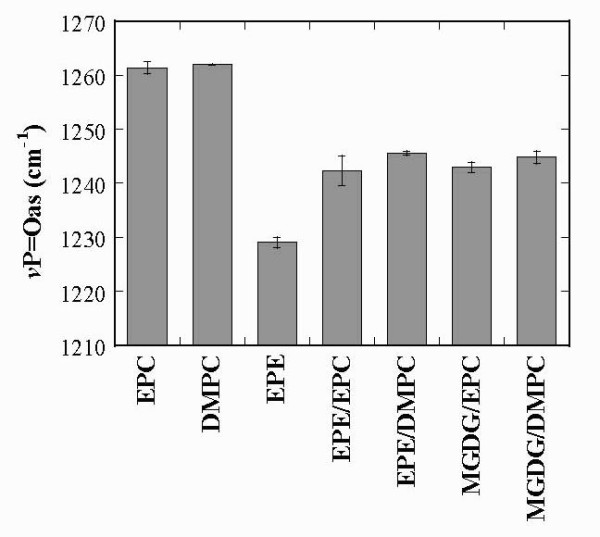
**νP=Oas peak positions determined from dry samples containing the indicated lipid compositions (compare Fig. 3)**. The values represent the means ± SE from at least 3 different samples.

Figure 5 shows the positions of the P=O peaks of the dry samples. For EPC and DMPC liposomes the P=O peak was situated at 1262 cm^-1^, clearly indicating that the samples in the vacuum cuvette of the FTIR spectrometer were anhydrous. For pure EPE the peak was situated at 1230 cm^-1^, indicating massive H-bonding between P=O and ethanolamine groups, in agreement with earlier reports [9]. In EPE/EPC and EPE/DMPC bilayers the P=O peak showed an intermediate position between the pure lipids due to both a reduction in EPE content and a shift of H-bonding of ethanolamine groups from P=O to C=O groups, as described above. Similar intermediate positions were observed for the P=O peak of MGDG/EPC and MGDG/DMPC liposomes. Obviously, no P=O peaks were present in the spectra from pure MGDG, as this galactolipid does not contain P=O groups. For liposomes containing PC and galactolipid the downfield shift of the P=O peak position in comparison with pure EPC and DMPC could be expected, as the galactose residues in MGDG should show a similar H-bonding behavior as those in digalactosyldiacylglycerol (DGDG) [14,56]. Interestingly, the positions of the P=O peaks of all mixed dry liposomes containing EPE or MGDG are very similar, indicating that the strength and/or amount of H-bonds between P=O groups and the ethanolamine or galactose moieties are comparable.

**Figure 6:**
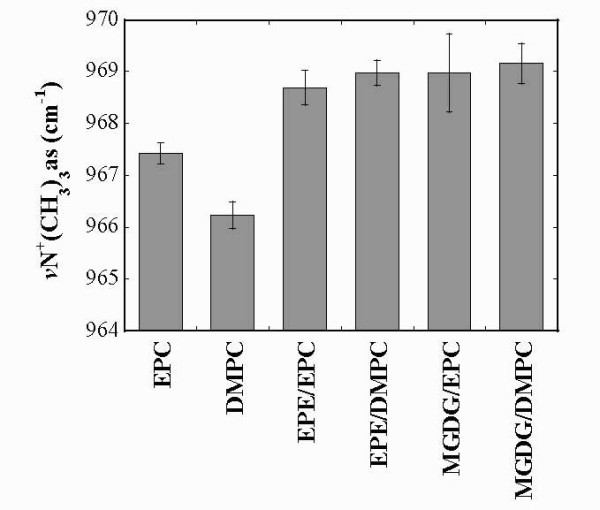
**νN^+^(CH_3_)_3_as peak positions determined from dry samples containing the indicated lipid compositions (compare Fig. 3)**. The values represent the means ± SE from at least 3 different samples.

The terminal part of the PC headgroup is the choline group with a characteristic asymmetric stretching vibration at around 970 cm^-1 ^which is sensitive to interactions with water [39,57] and sugars [40]. When the choline group is involved in interactions with water its vibration is shifted upfield by a maximum of 4 cm^-1 ^[39]. Figure 6 shows the positions of νN^+^(CH_3_)_3_as of dry liposomes containing either EPC or DMPC. The choline peak of EPC is centered at 967.5 cm^-1^, while for DMPC this peak is situated 1 cm^-1 ^lower, again indicating restrictions of the interactions in more tightly packed bilayers. In all mixed liposomes the choline peak was shifted to higher wavenumbers compared to pure PC, indicating increased interactions. Similar to the P=O peak in mixed membranes, the shift in the choline peak in the presence of EPE or MGDG was of similar magnitude.

**Figure 7:**
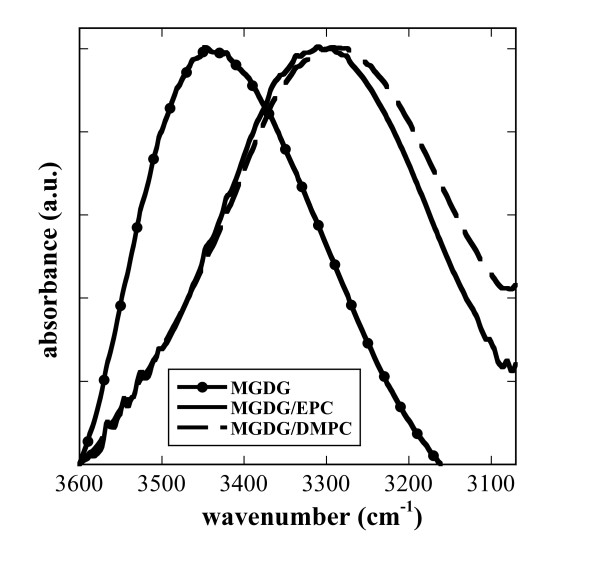
**Infrared spectra in the OH stretching vibration (νOH) region of the galactose headgroups of MGDG in pure MGDG and the indicated binary mixtures**.

**Figure 8:**
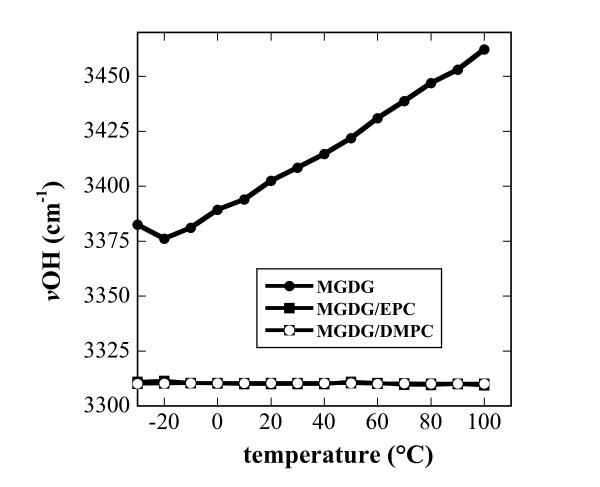
**νOH of the galactose headgroups of MGDG in pure MGDG and in the indicated binary mixtures (compare Fig. 7) as a function of temperature**. The peak positions determined from the two binary mixtures were almost identical at all temperatures and therefore only the symbols for one lipid composition are visible.

MGDG contains a galactose moiety that is able to H-bond to other headgroups through the sugar OH groups. The νOH presents a broad peak in the FTIR spectrum from about 3600 cm^-1 ^to 3100 cm^-1 ^(Figure 7), indicating a wide variety of H-bonding lengths and strengths. In pure, dry amorphous sucrose νOH is centered at 3370 cm^-1 ^and this band is shifted to about 3320 cm^-1 ^for sucrose fully H-bonded to EPC membranes in the dry state [40,58]. In pure, dry MGDG νOH was centered at 3448 cm^-1 ^(Figure 7), indicating only a low degree of H-bonding between MGDG molecules. In membranes containing 50% of either EPC or DMPC, νOH was shifted by more that 100 wavenumbers to around 3310 cm^-1^, emphasizing the strong intermolecular H-bonding between MGDG and PC noted above (Figure 4 and 5). The exceptional strength of these bonds is also indicated by the complete absence of a temperature dependence in νOH in the mixed membranes, while the low level of H-bonding in pure MGDG was further decreased at higher temperatures (Figure 8).

## Discussion

In the present paper we used both FTIR spectroscopy and DSC to determine the phase behavior and the molecular interactions of bilayer and nonbilayer lipids. This combination was necessary, as none of the methods alone would have yielded a complete picture of these dry lipid systems. The position of the νCH_2_s peak in FTIR spectra is sensitive to the mobility of the fatty acyl chains and can thus be used to discriminate between lipids in the gel state and lipids in either the liquid-crystalline or nonlamellar state [43]. Consequently, only transitions between gel phase and either of the other two phases were detected, but not transitions from liquid-crystalline to nonlamellar. In addition to the gel phase, fully saturated lipids such as DMPC can also form a solid (crystalline) phase [59,60]. However, we found no evidence of an additional low-temperature phase transition in either the FTIR or DSC data. In DSC thermograms gel to liquid-crystalline and liquid-crystalline to nonlamellar transitions can be easily distinguished due to the large difference in transition enthalpy [7]. However, with DSC interactions between lipid molecules can not be detected, making a combination of these two methods especially useful for the characterization of interactions between lipids and between lipids and interacting solutes or proteins. It should, however, be noted that neither method allows absolute phase assignments, but only records transitions between phases.

Pure, dry EPE showed one phase transition in the FTIR, corresponding to a gel to liquid-crystalline transition and an additional low enthalpy transition to H_II _was detected by DSC. This sequence of transitions has also been reported for different PEs in the hydrated state [20,61-63]. The T_m _for pure EPE was significantly lower than that determined by DSC for pure dry POPE (73°C, [60]). In addition, the same T_m _was found for dry POPE and POPC, while we found a large difference in the T_m _of dry EPE and EPC. These differences have to be attributed to the different fatty acid composition of the lipids, as EPE and EPC are not composed of identical fatty acids (compare Methods section).

Due to the high degree of unsaturation of the fatty acyl chains of the MGDG from spinach leaves used in this study and the resultant high mobility of the chains, νCH_2_s was located at high wavenumbers (above 2854 cm^-1^) even at -30°C and there was no indication for a phase transition in the FTIR data. There was only one low enthalpy phase transition apparent in the DSC thermograms, indicating that dry MGDG was in the liquid-crystalline phase at low temperature and in a nonlamellar phase at high temperature. For fully hydrated MGDG previous DSC measurements have shown a transition from H_II _to lamellar phase at -30°C during cooling [26] and a lamellar to H_II _transition at -44°C during heating [16].

In all mixed bilayer systems (EPE/EPC, EPE/DMPC, MGDG/EPC, MGDG/DMPC) only gel to liquid-crystalline phase transitions were detected by FTIR and DSC, while the DSC thermograms indicated that the transition from liquid-crystalline to nonbilayer was completely abolished. As expected, T_m _was significantly higher in membranes containing DMPC than in those containing EPC. Only in the case of MGDG/DMPC membranes, there was an indication for a partial demixing of the two lipids during the phase transition. This was only apparent in the DSC thermograms, where two peaks were resolved, but not in the FTIR melting curves that only showed one transition. We have shown previously that the resolution of more than one lipid melting event is possible with our FTIR method [53]. This suggests that the more unsaturated MGDG started to melt at a lower temperature than DMPC, but that the two melting events were too close together to be resolved by FTIR. In the liquid-crystalline state, the two lipids were completely mixed again, thereby preventing a further transition to H_II_, that was observed in pure MGDG. Interestingly, EPE/EPC membranes showed a phase transition that was almost identical to that of EPE (and not halfway between EPE and EPC), indicating the importance of headgroup interactions for the phase behavior of this lipid mixture.

In the fully hydrated state it has been shown previously for MGDG/DMPC [64], EPE/EPC, DOPE/DOPC [22] and DMPC/DMPE [11] that stable bilayers were formed, where the two lipids were well mixed and did not form separate domains. On the other hand, it has been suggested that during dehydration, when the formation of nonlamellar structures is favoured, phase separation of lipids with different phase preferences can take place, leading to freezing and dehydration induced membrane damage [32,65]. The results presented here suggest that for the investigated lipid mixtures such phase separation and H_II _formation did not occur during drying.

FTIR has provided evidence for complex interactions between lipid headgroups in the dry state that was observed at the C=O (all lipids), P=O (EPE, EPC, DMPC), choline (EPC, DMPC) and sugar OH (MGDG) levels. These interactions are made possible by the strong tilt of the headgroups in PC [66] and PE [67] towards the membrane surface in the hydrated state, which is increased during dehydration, as shown for PC [68]. For MGDG, some results also suggest a headgroup orientation almost parallel to the surface of the lipid bilayer [28], while other studies indicated that β-anomeric linkages of monosacharides, as in MGDG, lead to an orientation of the sugar away from the surface of fully hydrated membranes [69]. The strong involvement of the MGDG headgroup in H-bonding interactions that we found in the present study indicates that at least in the dry state the headgroup is bent towards the membrane surface, similar to the DGDG headgroup [70]. A comparison of the interaction patterns of the different functional groups across all relevant lipid combinations yielded results consistent with this assumption.

The ethanolamine in PE has been shown previously to form a network of strong H-bonds and electrostatic interactions with P=O groups [9,63,71]. These interactions result in a relatively low frequency of the νP=Oas vibration compared to PC. In the dry state νP=Oas of DMPC is situated at 1262 cm^-1^, while in the fully hydrated state it is shifted to around 1220 cm^-1 ^[52]. For hydrated DMPE the frequency of the P=O vibration was found at 1217 cm^-1 ^[72,73], while for dry PE values between 1231 cm^-1 ^and 1234 cm^-1 ^[74] were reported, in good agreement with the value of 1230 cm^-1 ^measured here for EPE.

The νP=Oas of mixed membranes containing EPE/EPC or EPE/DMPC had intermediate positions, in agreement with recent data on hydrated EPE/EPC mixtures [11]. The spectra also indicate that the interactions of ethanolamine groups were limited to the P=O groups and that access to the C=O groups was strongly restricted. This was reflected in the position of the C=O peak, which was highest in pure EPE and in the A C=O_H-bonded_/A C=O_free _ratio, which also showed the lowest value for pure EPE. In the mixed membranes, this ratio increased, indicating increased H-bonding of the ethanolamine groups to the C=O groups with a concomitant decrease in the interactions with the P=O groups. A similar increase was also observed in H-bonding to the choline groups, in agreement with a general redistribution of the interaction pattern between pure EPE and EPE/EPC mixtures. H-bonding to the C=O groups was strikingly lower in membranes containing DMPC than in those containing EPC, indicating that the tight packing of the membranes in the presence of a fully saturated lipid restricted access of other groups to the C=O moieties.

The OH groups in the galactose headgroup of MGDG H-bond effectively to the C=O, P=O and choline groups. In pure MGDG the fraction of C=O groups involved in H-bonding was more than twice that of free C=O and also the mixtures with PCs showed high A C=O_H-bonded_/A C=O_free _ratios. Similarly, these mixtures showed reduced νP=Oas and increased νN^+^(CH_3_)_3_as values in comparison to pure EPC and DMPC, indicating H-bonding with the galactose OH groups. Additional evidence for these H-bonding interactions was found in the massive shift of the νOH vibration by more than 100 wavenumbers in the mixed membranes compared to pure MGDG. A similar pattern of H-bonding has previously been shown in dry samples of mixed EPC/DGDG liposomes [39].

While the H-bonding with the C=O groups in the mixed membranes was always weaker in the presence of DMPC than in the presence of EPC, the degree of lipid unsaturation was less important for interactions with the P=O groups. The phosphate group is situated further away from the hydrophobic core of the membrane than the carbonyl esters and therefore the packing of the acyl chains and the area per lipid molecule have less influence on the degree of interaction.

## Conclusions

We have provided a comprehensive characterization of dry membrane systems containing two biologically important nonbilayer lipids that will help in the future to evaluate mechanisms and consequences of the interactions of solutes and proteins that may be involved in cellular desiccation tolerance. In particular, the detailed FTIR analyses of the interfacial and headgroup regions of these membrane systems will enable a detailed understanding of the mode of action of such protective molecules that may play crucial roles in anhydrobiosis [75], still one of the most enigmatic phenomena in biology.

## Authors' contributions

AVP designed and conducted all experiments, analyzed the data and drafted the manuscript. DKH participated in experimental design and data analysis, and wrote the final manuscript. Both authors read and approved the final manuscript.
